# Effects of Mitoquinone (MitoQ) Supplementation on Aerobic Exercise Performance and Oxidative Damage: A Systematic Review and Meta-analysis

**DOI:** 10.1186/s40798-024-00741-5

**Published:** 2024-07-09

**Authors:** Oliver Gonzalo-Skok, Rafael A. Casuso

**Affiliations:** 1 Department of Communication and Education, Faculty of Physical Activity and Sports, Universidad Loyola Andalucía, Sevilla, Spain; 2 Department of Health Sciences, Faculty of Physical Activity and Sports, Universidad Loyola Andalucía, Córdoba, Spain

**Keywords:** Training, Oxidative Stress, Ergogenic, Mitochondria

## Abstract

**Background:**

Contracting skeletal muscle produces reactive oxygen species (ROS) originating from both mitochondrial and cytosolic sources. The use of non-specific antioxidants, such as vitamins C and E, during exercise has produced inconsistent results in terms of exercise performance. Consequently, the effects of the mitochondrial-targeted coenzyme Q10, named Mitoquinone (MitoQ) on exercise responses are currently under investigation.

**Methods:**

In this study, we conducted a meta-analysis to quantitatively synthesize research assessing the impact of MitoQ on aerobic endurance performance and exercise-induced oxidative damage. PubMed, Web of Science, and SCOPUS databases were used to select articles from inception to January 16th of 2024. Inclusion criteria were MitoQ supplementation must be compared with a placebo group, showing acute exercise responses in both; for crossover designs, at least 14 d of washout was needed, and exercise training can be concomitant to MitoQ or placebo supplementation if the study meets the other inclusion criteria points. The risk of bias was evaluated through the Critical Appraisal Checklist (JBI).

**Results:**

We identified eight studies encompassing a total sample size of 188 subjects. Our findings indicate that MitoQ supplementation effectively reduces exercise-induced oxidative damage (SMD: -1.33; 95% CI: -2.24 to -0.43). Furthermore, our findings indicate that acute and/or chronic MitoQ supplementation does not improve endurance exercise performance (SMD: -0.50; 95% CI: -1.39 to 0.40) despite reducing exercise-induced oxidative stress. Notably, our sensitivity analysis reveals that MitoQ may benefit subjects with peripheral artery disease (PAD) in improving exercise tolerance.

**Conclusion:**

While MitoQ effectively reduces exercise-induced oxidative damage, no evidence suggests that aerobic exercise performance is enhanced by either acute or chronic MitoQ supplementation. However, acute MitoQ supplementation may improve exercise tolerance in subjects with PAD. Future research should investigate whether MitoQ supplementation concurrent with exercise training (e.g., 4–16 weeks) alters adaptations induced by exercise alone and using different doses.

**Supplementary Information:**

The online version contains supplementary material available at 10.1186/s40798-024-00741-5.

## Key points

Contracting skeletal muscle produces ROS from both mitochondrial and cytosolic sources, potentially impairing exercise performance.

MitoQ supplementation reduces exercise-induced oxidative damage but does not improve endurance exercise performance.

**Supplementary Information:**

The online version contains supplementary material available at 10.1186/s40798-024-00741-5.

## Background

Oxidative metabolism efficiently generates significant energy by completely oxidizing glucose and fatty acids within mitochondria [1]. This process involves an enzymatic cascade that produces reactive oxygen species (ROS), such as singlet oxygen, hydrogen peroxide, and the hydroxyl radical. Excess ROS production is associated with pathophysiological conditions, including insulin resistance and cardiovascular diseases [2–4]. Additionally, the overproduction of ROS can induce muscle fatigue during intense contractions [5, 6] and lead to oxidative damage in cellular compartments, proteins, and lipids [7]. Exercise adaptations include increased ROS-detoxifying enzymes, enhancing antioxidant capacity [8], and modifications within the mitochondrial electron transport chain to minimize ROS production [9]. These adaptations decrease ROS bursts, maintaining the redox state within physiological levels [10]. Importantly, ROS serve as critical signaling factors, mediating metabolic and physical adaptations to exercise [11, 12]. Thus, balancing ROS signaling and preventing oxidative damage is essential. However, non-specific antioxidants like vitamins C and E have not demonstrated benefits superior to placebo regarding exercise adaptations or responses [13–16].

Studies on the supplementation with antioxidant vitamins C and E have shown that they specifically prevent the mitochondrial adaptations induced by exercise [11, 12]. However, the mechanism remains unclear, as these antioxidants appear to block the entire pathway, from nuclear transcription factors to the expression of the electron transport chain machinery and mitochondrial DNA [11, 12]. Contracting skeletal muscle generates both mitochondrial and cytosolic ROS. The primary sources of mitochondrial ROS are complexes I and III of the electron transport chain [17], whereas the main cytosolic source is NADPH oxidase isoform 2 (NOX2) [18]. It should be noted that during exercise, ROS production from Complex I and Complex III is reduced to below basal levels [17]. Which may suggest that reducing mitochondrial ROS during exercise could be a desirable target to maintain muscle homeostasis. Notably, NOXs-related ROS production is crucial in key exercise events such as glucose uptake and calcium kinetics [18, 19]. In addition, ROS production by NOX enzymes appears to mediate exercise adaptations, including improvements in mitochondrial function [20] and insulin sensitivity [21]. Consequently, a novel research topic in exercise sciences is supplementing with a mitochondrial-targeted antioxidant during exercise to reduce excessive mitochondrial ROS while preserving cytosolic ROS production.

Coenzyme Q supplementation as an antioxidant has seen limited success due to its lipophilic nature which limits its bioavailability [22]. In contrast, mitoquinone (MitoQ) is an orally available mitochondrial-targeted coenzyme Q variant [23]. Mitoquinone, a conjugate of coenzyme Q, effectively positions the quinone moiety within the hydrophobic core of the polarized IMM, making MitoQ an efficient superoxide scavenger that attenuates lipid peroxidation [24]. Moreover, as training is known to increase Coenzyme Q_10_ levels [25, 26] which may enhance mitochondrial function [27]. There is growing interest in the effects of MitoQ supplementation on exercise performance. However, studies have yielded mixed results; some report improvements in performance [28], while others find no significant effects [29, 30]. Nevertheless, no study has already synthetized the availably data to reach a solid conclusion. Therefore, our study aims to quantitatively summarize, through a meta-analysis of randomized trials, the effects of MitoQ supplementation on aerobic exercise performance and exercise-induced oxidative damage.

## Methods

### Data Search and Study Selection

The Preferred Reporting Items for Systematic Reviews and Meta-Analyses (PRISMA) 2020 statement was used to report the items of research studies used [31]. The protocol was registered in the International Prospective Register of Systematic Reviews (PROSPERO) database (CRD42023477400). The authors systematically searched for eligible articles in PubMed, Web of Science, and SCOPUS databases from inception to January 16th of 2024. To be considered, the studies must be published in English or Spanish and performed on adult humans (≥ 18 year.). Inclusion criteria: (1) MitoQ supplementation must be compared with a placebo group; for crossover designs, at least 14 d of washout was needed; (2) acute exercise-induced oxidative damage and/or exercise performance must be reported for both placebo and MitoQ groups; (3) exercise training can be concomitant to mitoQ or placebo supplementation if the study meets the other inclusion criteria points. Exclusion criteria: concomitant supplementation with other(s) antioxidants and studies not performed in humans. As the present topic is relatively recent, we performed a broad search strategy as follows: (“exercise” or “training” or “sport”) and (“mitoquinone” or “mitoquinol” or “mitoQ” or “mitochondrial-targeted antioxidant” or “mitochondrial targeted antioxidant” or “mitochondria-targeted coenzyme Q10” or “mitochondria-targeted coenzyme Q10”). To identify missing studies, each selected study was individually scrutinized by clicking on the “cited” and “similar” tabs of the databases. The two authors independently selected the studies. Studies were screened based on their titles and abstracts. Discrepancies that arose during the study selection were resolved by consensus after discussion.

### Data Extraction, Synthesis, and Analysis

The two researchers independently extracted the following data: number of subjects in each group, sex, age, body mass index (BMI), weight, height, health status, plasma oxidative stress markers, and aerobic endurance performance markers. If data were not presented in the text or tables, data were extracted using WebPlotDigitalizer [32]. The mean and standard deviation (ΔSD) change was recorded for each treatment (i.e., MitoQ and Placebo) and outcome. One study [29] did not report preliminary data, so changes were not reported. As this was a crossover trial, we collected only the post-Placebo and post-MitoQ time to exhaustion data, and the effect size was calculated using the post-Placebo mean (SD) and the post-MitoQ mean (SD). We used a similar approach previously when both sets of data came from the same subjects [33, 34]. Additionally, a sensitivity analysis was used to confirm that this approach did not affect the overall results. When ΔSD was not reported we calculated it assuming a correlation coefficient of 0.7 as previously suggested [35, 36]:

$$\begin{array}{l}{\rm{\Delta SD = }}\\\sqrt {\left( {{\rm{S}}{{\rm{D}}_{{\rm{pre}}}}^{\rm{2}}{\rm{ + S}}{{\rm{D}}_{{\rm{post}}}}^{\rm{2}} - {\rm{2}}\,{\rm{ \times }}\,{\rm{corr}}\,{\rm{ \times }}\,{\rm{S}}{{\rm{D}}_{{\rm{pre}}}}\,{\rm{ \times }}\,{\rm{S}}{{\rm{D}}_{{\rm{post}}}}} \right)} \end{array}$$

Regarding aerobic endurance performance, two studies performed time trials [37, 38] and two performed time to exhaustion trials [29, 39]. In this regard, we included the study by Shill et al. [30] in the analysis of aerobic exercise performance as it reported VO2max during a graded exercise test.

All analyses were performed using the meta for package of R software [40]. The meta-analyses were performed using random-effects models with DerSimonian‒Laird methods to assess the effects of MitoQ on exercise-induced oxidative stress and aerobic exercise performance. Effect sizes are presented as standardized mean differences (SMDs) and 95% CIs, as the outcomes have noncomparable scales.

### Risk of Bias and Heterogeneity

The Critical Appraisal Checklist for Randomized Controlled Trials of the Faculty of Health and Medical Sciences at the University of Adelaide, South Australia [41] was used to evaluate study bias. The checklist comprises 13 items relating to the article’s title, abstract, introduction, methods, results, and discussion sections. Quality scores were calculated as the total points and percentage of the applicable items. The overall quality was considered medium when the score ranged from 50 to 75% and high for it was *>* 75% [33]. Heterogeneity was reported as the Tau (*τ*)^2^ value which is an estimate of between-studies variance, and the confidence intervals was derived from *τ*.

Publication bias was assessed using visual inspection of Funnel plots and accompanying Egger’s Tests. Publication bias pertains to the tendency of significant results to be more likely published than null results. A *p*-value less than 0.05 in the Egger test indicates publication bias. As a sensitivity analysis, we employed the leave-one-out method to assess whether any of the included studies significantly influenced the overall effect. If the leave-one-out test yielded positive results, we reported the effect size of the model with that particular study excluded from the analysis.

## Results

**Fig. 1:**
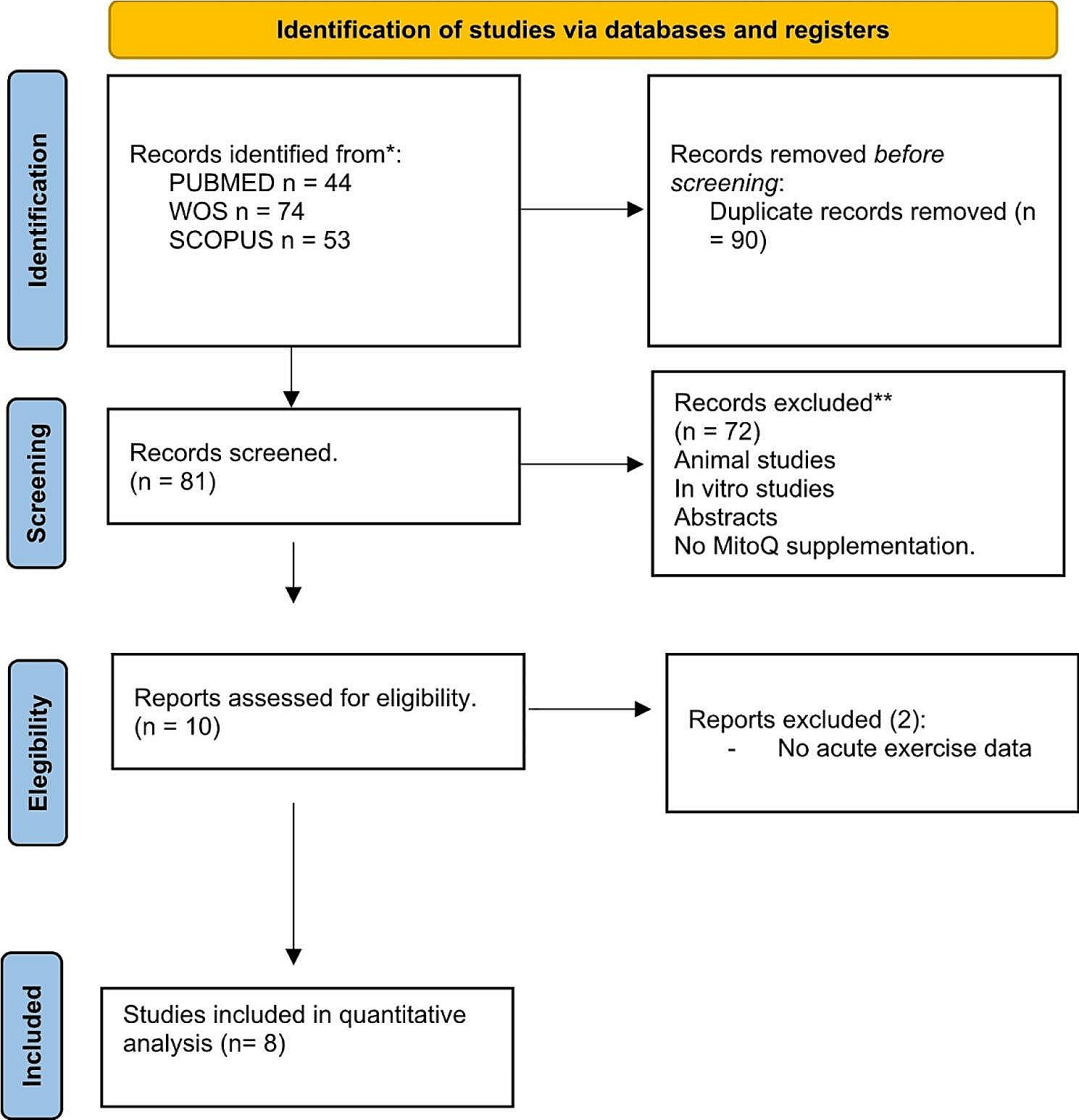
PRISMA flowchart

After deduplication, a total of 81 studies were screened (Fig. 1). Eight studies (involving 188 subjects) met the inclusion criteria and were included in the meta-analysis (Fig. 1). Two studies [42, 43] evaluated the effects of MitoQ supplementation and MitoQ in conjunction with training on basal blood redox state. However, since they did not analyze the response to an acute exercise, they were excluded from the analysis. Six of the included studies assessed healthy recreational subjects [28–30, 37, 38, 44], one study involved a population with peripheral artery disease (PAD) [39] and another study involved subjects with chronic kidney disease [45]. Six of these studies examined the effects of chronic supplementation (with an average duration of 28 days, ranging from 10 to 42 days), and three studies investigated the effects of acute supplementation [29, 39, 44] (Table 1). The risk of bias analysis of the studies indicated medium to high quality (Suppl Table 1).

**Table 1:** Study characteristics

Reference	Subjects	Protocol	Supplementation	Results	Comments
Hughes, 2023 [29]	9 physically inactive females Age: 47 ± 22 yr BMI: 28.5 ± 3.8 kg/m^2^	*Design*: Acute double-blind, randomized cross-over (Placebo and MitoQ). After reached a RER of > 1.0 (2 min rest), a protocol of 30 s at 30 W and 30 s at 100 W followed by 10 W increments every 1 min until exhaustion.	80 mg	No significant differences in submax metabolic nor cardiovascular variables between trials. VO_2max_ was higher during Placebo compared to Mito Q.	
Kirkman, 2023 [45]	18 patients with CKD (3 females) Age: 62 (32–78) BMI: 28.5 ± 3.8 kg/m^2^	*Design*: Randomized doble blind controlled parallel design (Placebo and MitoQ). Subjects were supplemented for 28 days. Exercise was assessed during an incremental test to fatigue in a cycle ergometer.	20 mg/d	No effect of MitoQ on exercise performance. MitoQ enhanced microvascular function.	
Broome, 2022 ii [37]	32 healthy males Age: 27 ± 4.5 yr BMI: 24.1 ± 3.1	*Design*: double-blind, parallel placebo-controlled Following supplementation (14d) subjects performed maximal eccentric contractions) 20 sets x 15 repetitions with 30 s between sets.	20 mg/d	Peak eccentric torque loss recovery was prevented in MitoQ. No differences between Pla and MitoQ on exercise-induced F2- isoprostanes. MitoQ showed increased CK levels.	
Broome, 2022 [38]	23 healthy males Age: 44 ± 7.8 yr BMI: 26 ± 3	*Design*: double-blind, placebo-controlled parallel. *Protocol*: Time trial + 10 days MitoQ/Pla supplementation + HIT trial + 3 weeks of training + Time trial	20 mg/d	MitoQ did not altered basal levels of oxidative stress /antioxidant markers. Circulating protein carbonyls was similar after exercise in Placebo and MitoQ. MitoQ improved the adaptations to exercise (peak power at VO2peak)	Supplementation was not maintained during the 3 weeks of training.
Broome, 2021 [28]	19 recreational male cyclists Age: 44 ± 4 year. BMI: 24.7 ± 3	*Design*: Double-blind, placebo-controlled crossover. *Protocol*: 28/d MitoQ/Pla + 8Km Time trial + 6-week washout + 28d MitoQ + 8Km time trial.	20 mg/d	MitoQ improved time trial performance. MitoQ increased blood lactate and reduced F2-isoprostanes in response to intense exercise.	The time trial was preceded by 45 min at 70% VO2max.
Williamson, 2020 [44]	24 healthy males	*Design*: double-blind, randomized, parallel placebo-controlled. *Acute phase*: MitoQ was consumed 1 h before high intensity exercise. *Chronic phase*: consisted of 21 days of supplementation and the exercise test was repeated after it.	20 mg/d	Blood hydroperoxides increased after exercise in both MitoQ and Pla either after acute or chronic supplementation.	HIT consisted in 4 × 4 min at 90–95% their maximal heart rate interspersed by 3 min of active recovery. Blood was collected before and after each exercise test.
Park, 2020 [39]	11 patients (5 women) with PAD Age: 66.1 ± 10.6 yr BMI: 30 ± 6.7	*Design*: Randomized, placebo-controlled, crossover. An acute dose of MitoQ/Pla was administered, and cardiovascular, metabolic, and physical function was assessed after 1 h.	80 mg/d	Mito Q increased maximal walking time and distance. Mito Q decreased MDA levels after exercise. Mito Q tended to reduce diastolic blood pressure.	
Shill, 2016 [30]	20 healthy men 22.1 ± 0.7 yr 26.9 ± 0.9 yr	*Design*: Randomized double blind parallel. For 3 weeks subjects trained and concomitantly received MitoQ/Pla.	10 mg/d	Mito Q did not further increased VO2max or reduced blood lipid peroxidation.	

Note: BMI: body mass index; CK: creatine kinase; CKD; chronic kidney disease; HIT: high-intensity training; PAD; periphery arterial disease; Pla: placebo; RER: Respiratory exchange ratio; VO_2max_: maximal oxygen consumption; VO_2peak_: peak of oxygen consumption; W: watts; yr: year

### Effect of MitoQ Supplementation on Aerobic Performance

**Fig. 2:**
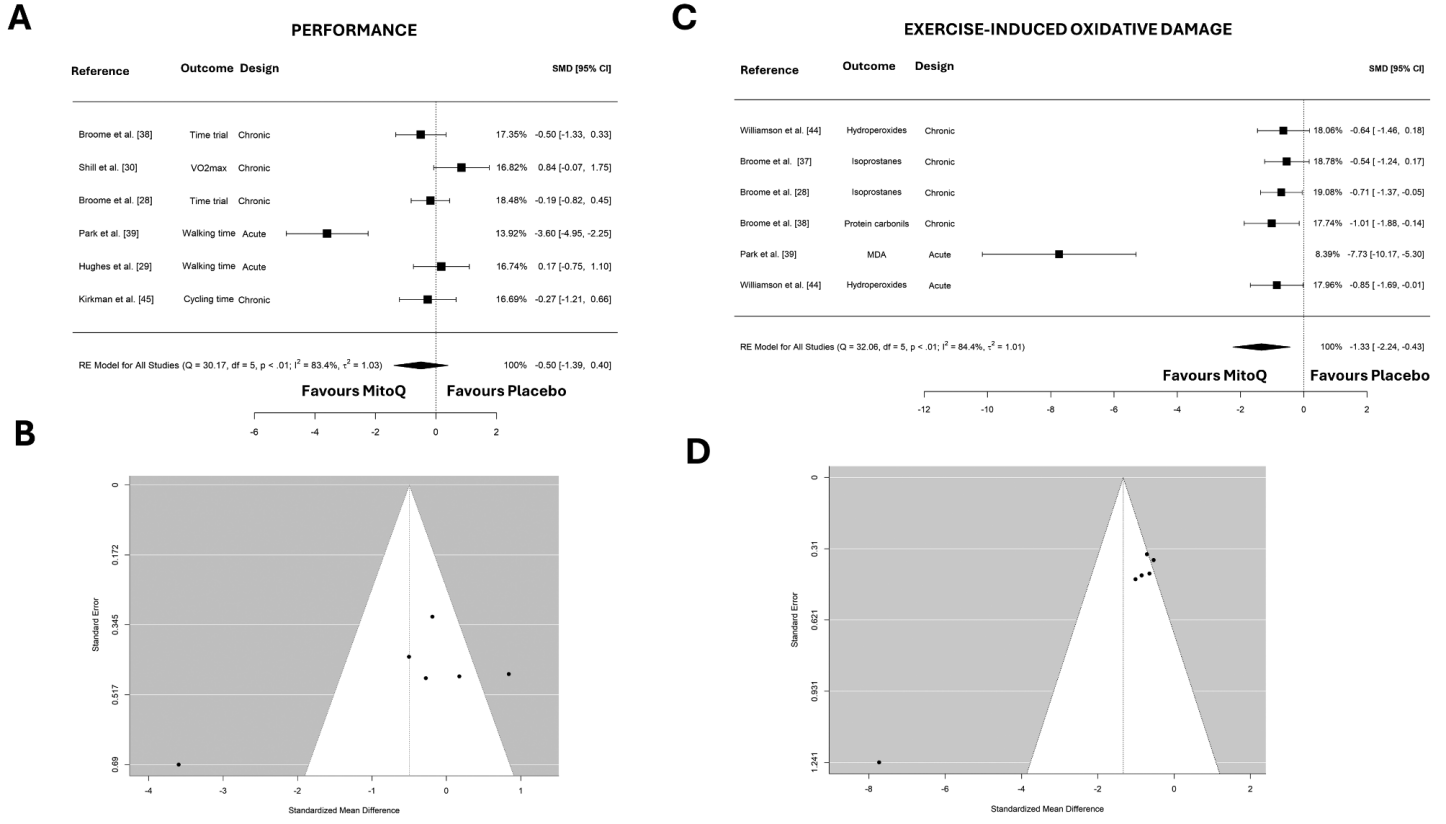
Forest plots of the effects of MitoQ on aerobic endurance performance (**A**), and exercise-oxidative damage (**C**). Panels **B** and **D** show the funnel plots for performance and oxidative stress respectively. MDA, malondialdehyde, RE, random effect; SMD, standardized mean difference

The effects of MitoQ supplementation on aerobic performance assessed in five studies (Fig. 2A). We found that MitoQ did not improve aerobic exercise performance (SMD: -0.50; 95% CI: -1.39 to 0.40; *τ*^2^ = 1.03; *p* = 0.398). Regarding publication bias, the Egger test yielded a non-significant result (*p* = 0.239, Fig. 2B). The sensitivity analysis revealed that the study by Park et al. [39] influenced the overall effects. When excluding this study from the analysis, the result yielded a similar result (SMD: -0.03; 95% CI: -0.46 to 0.41; *τ*^2^ = 0.06; *p* = 0.909).

### Effect of MitoQ Supplementation on Exercise-induced Oxidative Damage

Exercise-induced oxidative stress was evaluated in six studies (Fig. 2C). We found that MitoQ supplementation decreased blood markers of oxidative damage (SMD: -1.33; 95% CI: -2.24 to -0.43; *τ*^2^ = 1.01; *p* = 0.004). Regarding publication bias, the Egger test yielded a significant result (*p* = 0.002, Fig. 2D). At the same time, the sensitivity analysis revealed that the study by Park et al. [39] was influencing the overall effects. When excluding this study from the analysis, the result yielded a more robust result (SMD: -0.7; 95% CI: -1.07 to -0.39; *τ*^2^ = 0.00; *p* < 0.001).

## Discussion

Muscle contractions, such as those induced by exercise, increase ROS production. In this response, NOX enzymes are known to be a primary source of ROS, which likely facilitates performance by increasing glucose uptake [18]. On the other hand, although mitochondrial ROS production is limited during high-intensity exercise compared to NOX sources, it may still be sufficient to induce oxidative damage [44]. Therefore, our meta-analysis aimed to synthesize quantitatively the studies assessing the effects of the mitochondrial-targeted antioxidant MitoQ on aerobic endurance performance and exercise-induced oxidative damage.

The main finding of our work is that MitoQ supplementation does not enhance aerobic exercise performance. The main potential explanation for this phenomenon is that mitochondrial ROS production can decrease during exercise, even falling below resting levels [17]. Thus, targeting mitochondrial ROS production may have limited effects during exercise. Additionally, it is now known that several CoQ pools exist in mammalian mitochondria [46]. When glycolysis is the main source of electrons, they primarily pass from NADH to Complex I (CI) to Complex III (CIII) through a CI-linked Q-pool [47]. However, when β-oxidation predominates, the relative amount of FADH_2_​ increases, and electrons are mainly taken up by a free Q-pool linked to CIII [47]. Failure to modulate the available CoQ pools according to physiological needs decreases maximal exercise capacity in rodents [48]. It has been suggested that fatty acid-linked respiration increases more than CI and Complex II (CII)-linked respiration after an exercise period [49]. Therefore, muscle mitochondria adapt strongly to take electrons from FADH2​ to a Q-pool dedicated to CIII. Thus, it would be logical that higher CoQ levels, achieved by intake of Q analogs such as MitoQ, may help channel electrons to CIII. However, when MitoQ is supplemented during exercise, there are no improvements in muscular oxidative capacity [30]. Moreover, 20 days of MitoQ supplementation does not increase mitochondrial content [38], which can have significant implications if CIII (and/or related dehydrogenases) does not increase correspondingly with CoQ levels, as reductive stress can arise [50]. Therefore, the accumulation of reducing equivalents can explain the unchanged exercise performance while preventing oxidative stress. In addition, this situation can also trigger long-term CI degradation via reverse electron transport [47], which would be counterproductive for maximal exercise performance. In the future, studies addressing MitoQ in muscle physiology should consider assessing electron transport chain complexes as well as mitochondrial respiration using different substrates.

Our sensitivity analyses identified the study by Park et al. [39] as a source of heterogeneity. This study was conducted in subjects with PAD and found that an acute 80 mg dose of MitoQ, administered one hour before an exercise test, improved walking time by 17% compared to placebo. It also enhanced flow-mediated dilation in the legs, contrasting with another recent study where an acute 80 mg dose of MitoQ reduced VO_2max_ and maximal ventilation in sedentary healthy subjects [29]. The latter study suggested that this effect might be due to an inhibition of exercise-induced pulmonary vasoconstriction. Another study examined the effects of 28 days of MitoQ supplementation in patients with chronic kidney disease [45]. Although aerobic exercise capacity was not improved, MitoQ supplementation enhanced microvascular vasodilation [45]. Taken together, these data suggest that MitoQ could potentially impact blood flow by increasing vasodilation. While this can be beneficial for patients with some degree of vascular dysfunction, in the context of training, it could elicit a negative response because local vasoconstriction is necessary during intense exercise. However, it should be highlighted that although patients with some degree of vascular dysfunction could benefit in terms of aerobic performance, this was only observed in patients with PAD [39], not in those with chronic kidney disease [45]. One possible explanation could be that the study by Kirkman et al. [45] assessed fatigue during progressive exercise on a cycle ergometer, where exercise limitation can be due to local rather than central fatigue. Additionally, these studies differed in the duration of supplementation, which was acute (1 h before exercise) in the study by Park et al. [39], and chronic (over 28 consecutive days) in the study by Kirkman et al. [45], which can likely exert different physiological effects.

The study by Park et al. [39] also contributed significantly to the heterogeneity among studies analyzing exercise-induced oxidative damage. ROS are known to act as crucial signaling molecules, regulating gene expression, enzyme activity, and membrane transport during exercise [51]. However, the ROS burst induced by exercise can surpass physiological limits, leading to significant oxidative damage in conditions with enhanced mitochondrial ROS production, such as in patients with PAD. This may also explain the observed improvement in walking time after MitoQ supplementation in PAD patients. Indeed, high-intensity muscle contractions can produce excessive ROS, potentially reducing muscle force generation [6]. In such scenarios, limiting ROS production or redirecting it to cytosolic sources might enhance endurance performance. However, it should be noted that the Park et al. [39] study employed the thiobarbituric Acid Reactive Substances (TBARS) assay to indirectly assess lipid peroxidation, a method reported to be a less reliable marker for detecting lipid peroxidation in response to exercise [52]. Therefore, the precise impact of MitoQ supplementation on excessive ROS production during exercise in subjects with altered mitochondrial function, like those with PAD, remains unclear. Additionally, the dose-response relationship of MitoQ merits further investigation, as varying dosages, such as the 20 mg used by Williamson et al. [44] versus the 80 mg used by Park et al. [39], may account for the significant differences observed in exercise-induced oxidative damage between these two studies employing an acute supplementation design. Nevertheless, it appears likely that similar doses, for instance, 80 mg, may have differing effects on exercise time to exhaustion depending on the health status of the subjects [29, 39].

### Limitations and Strengths

The main limitation of our study is the relative novelty of MitoQ supplementation in sports science, resulting in limited published research. Other limitations are the relatively small participant number, as well as the relatively short duration (i.e., acute and 3 weeks) of supplementation in published studies. In addition, there were only 17 females out of 188 participants and, thus, it should be considered as a limitation. Finally, we investigated oxidative stress biomarkers in plasma, which limits our ability to draw mechanistic conclusions about the effects of MitoQ supplementation on exercise-induced muscle oxidative stress. However, the novelty of this research topic also serves as a strength, as significant findings can guide the development of future studies. There is a clear need to investigate whether MitoQ supplementation concurrent with exercise training (e.g., 4–16 weeks) alters adaptations induced by exercise alone. Additionally, studies examining various doses, ranging from 10 to 80 mg, are warranted, as well as comparisons between non-specific antioxidants and MitoQ to discern the impact of ROS sources on exercise adaptations.

## Conclusions

We conducted the first meta-analysis of randomized trials exploring the effects of MitoQ on aerobic exercise performance and exercise-induced oxidative damage. Our findings indicate that while MitoQ effectively reduces exercise-induced oxidative damage, no evidence suggests that aerobic exercise performance is enhanced by either acute or chronic MitoQ supplementation. However, acute MitoQ supplementation may improve exercise tolerance in subjects with PAD. Investigating whether similar benefits extend to other diseases characterized by significant alterations in mitochondrial ROS production could be highly valuable.

### Electronic Supplementary Material

Below is the link to the electronic supplementary material.

Supplementary Material 1

## Data Availability

Data used for the present study is available from the corresponding author.

## Abbreviations

MitoQ: Mitoquinone
NOX: NADPH oxidase
PAD: Peripheral artery disease
ROS: Reactive oxygen species
SMDs: Standardized mean differences
