# Supplementary material for: Effects of Mitoquinone (MitoQ) Supplementation on Aerobic Exercise Performance and Oxidative Damage: A Systematic Review and Meta-analysis
Source: Sports Med Open. 2024 Jul 9;10:77. doi: 10.1186/s40798-024-00741-5 (PMC11233485; doi:10.1186/s40798-024-00741-5)
Supplement: Supplementary file 1 — Supplementary Material 1 [file 40798_2024_741_MOESM1_ESM.docx]

**Effects of mitochondrial-targeted antioxidant supplementation on aerobic exercise performance and oxidative damage: A systematic review and meta-analysis**

Oliver Gonzalo Skok^1^ and Rafael A. Casuso^2*^

^1^Universidad Loyola Andalucía, Department of Communication and Education, Faculty of Physical Activity and Sports, Sevilla (Spain). ^2^Universidad Loyola Andalucía, Department of Health Sciences, Faculty of Physical Activity and Sports, Córdoba (Spain).

*Corresponding Author: racasuso@uloyola.es

**Supplementary Table 1**. Risk of bias and quality assessment.

| **Study** | **1** | **2** | **3** | **4** | **5** | **6** | **7** | **8** | **9** | **10** | **11** | **12** | **13** | **Percentage reached** | **Quality level** |
| --- | --- | --- | --- | --- | --- | --- | --- | --- | --- | --- | --- | --- | --- | --- | --- |
| Shill et al. 2016 | 1 | 1 | 2 | 2 | 1 | 1 | 2 | 2 | 2 | 1 | 1 | 1 | 2 | 73% | MQ |
| Park et al. 2020 | 1 | 1 | 2 | 2 | 1 | 1 | 2 | 1 | 2 | 1 | 1 | 2 | 2 | 73% | MQ |
| Williamson et al. 2020 | 1 | 1 | 2 | 2 | 1 | 1 | 2 | 2 | 2 | 1 | 2 | 1 | 2 | 77% | HQ |
| Broome et al. 2021 | 1 | 1 | 2 | 2 | 2 | 2 | 2 | 1 | 2 | 2 | 2 | 1 | 2 | 80% | HQ |
| Broome et al. 2022 | 1 | 1 | 2 | 2 | 2 | 2 | 2 | 1 | 2 | 1 | 2 | 1 | 2 | 84% | HQ |
| Broome et al. 2022ii | 1 | 1 | 2 | 2 | 2 | 1 | 2 | 2 | 2 | 1 | 2 | 1 | 2 | 81% | HQ |
| Hughes et al. 2023 | 1 | 1 | 2 | 2 | 1 | 1 | 2 | 1 | 2 | 1 | 2 | 1 | 0 | 65% | MQ |
| Kirkman et al. 2023 | 1 | 1 | 2 | 2 | 1 | 1 | 2 | 1 | 2 | 1 | 1 | 2 | 2 | 73% | MQ |
|  |  |  |  |  |  |  |  |  |  |  |  |  |  |  |  |

Checklist from Joanna Briggs Institute's criterium. 0:No; 1: Unclear; 2: Yes. HQ: high quality; MQ: medium quality.
